# Role of Endogenous ENaC and TRP Channels in the Myogenic Response of Rat Posterior Cerebral Arteries

**DOI:** 10.1371/journal.pone.0084194

**Published:** 2013-12-31

**Authors:** Eok-Cheon Kim, Soo-Kyoung Choi, Mihwa Lim, Soo-In Yeon, Young-Ho Lee

**Affiliations:** Department of Physiology, College of Medicine, Brain Korea 21 PLUS Project for Medical Science, Yonsei University, Seoul, Korea; Osaka University Graduate School of Medicine, Japan

## Abstract

**Aims:**

Mechanogated ion channels are predicted to mediate pressure-induced myogenic vasoconstriction in small resistance arteries. Recent findings have indicated that transient receptor potential (TRP) channels and epithelial sodium channels (ENaC) are involved in mechanotransduction. The purpose of this study was to investigate the role of TRP channels and ENaC in the myogenic response. Our previous study suggested that ENaC could be a component of the mechanosensitive ion channels in rat posterior cerebral arteries (PCA). However, the specific ion channel proteins mediating myogenic constriction are unknown. Here we found, for the first time, that ENaC interacted with TRPM4 but not with TRPC6 using immunoprecipitation and confocal microscopy.

**Methods and Results:**

Treatment with a specific βENaC inhibitor, amiloride, a specific TRPM4 inhibitor, 9-phenanthrol, and a TRPC6 inhibitor, SKF96365, resulted in inhibition of the pressure-induced myogenic response. Moreover, the myogenic response was inhibited in rat PCA transfected with small interfering RNA of βENaC, TRPM4, and TRPC6. Co-treatment with amiloride and 9-phenanthrol showed a similar inhibitory effect on myogenic contraction compared to single treatment with amiloride or 9-phenanthrol. The myogenic response was not affected by 9-phenanthrol or amiloride treatment in PCA transfected with βENaC or TRPM4 siRNA, respectively. However, pressure-induced myogenic response was fully inhibited by co-treatment with amiloride, 9-phenanthrol, and SKF96365, and by treatment with SKF96365 in PCA transfected with βENaC siRNA.

**Conclusion:**

Our results suggest that ENaC, TRPM4, and TRPC6 play important roles in the pressure-induced myogenic response, and that ENaC and TRPM4 interact in rat PCA.

## Introduction

In many vessels, such as coronary, mesenteric, renal, and cerebral arteries, blood flow is tightly regulated despite changes in systemic perfusion pressure [1]–[4]. Within the physiological perfusion pressure range, small resistant arteries respond to an acute increase in pressure by vasoconstricting and to a decrease in pressure by vasodilating. This behavior, termed the myogenic response, is inherent to vascular smooth muscle in the vessel wall of small arteries and arterioles and is not dependent on the endothelium or the nervous system [2], [5], [6]. The myogenic response has been described in many vessels including cerebral, mesenteric, renal, femoral, and pulmonary arteries [7]–[11]. Alterations in the myogenic response have been shown to be associated with diseases such as hypertension and diabetes [12]. The functional importance of the myogenic response in health and disease had made it one of the most intensively investigated topics in circulatory physiology. Although the pressure-dependent myogenic response has been intensively investigated, the molecular mechanisms underlying the transduction of pressure into a cellular event in vascular smooth muscle cells (VSMCs) are still unclear.

Recent findings have indicated that two kinds of ion channels, transient receptor potential (TRP) channels and amiloride-sensitive epithelial sodium channels (ENaC) are involved in cellular mechanotransduction [13]–[20]. The most well-known role of ENaC is related to Na^+^ reabsorption in many epithelia, such as the kidney, distal colon, secretory glands, and respiratory airways. Because of their close evolutionary relationship to the *C. elegans* degenerins [21] and their requirement for normal mechanosensory responses, ENaC proteins are considered to be components of mechanosensitive ion channel complexes in vertebrate tissue.

Recently identified mammalian TRP channels are also candidates for mechanosensory functions in arterial smooth muscle [22]. Most TRP channels are non-selective cation channels permeable to calcium which have been implicated in a large variety of sensory functions [23]. Two members of the TRP family, TRPC6 and TRPM4, are considered to be mediators of pressure-induced myogenic constriction in cerebral vessels [13], [16]. ENaC [14], [19], TRPC6 [13], [24], and TRPM4 [15], [16] are expressed in rat cerebellar and posterior cerebral arteries (PCA). We also previously showed that the ENaC is critically involved in the cerebrovascular myogenic response. Thus, the goal of this study was to determine the roles of ENaC, TRPM4, and TRPC6 in the contractile response to pressure, and to investigate the functional relationships among ENaC, TRPM4, and TRPC6. We therefore examined the expression of ENaC, TRPC6, and TRPM4 in rat PCA. In addition, we analyzed the contributions of ENaC, TRPC6, and TRPM4 to the contractile response to pressure in the presence of pharmacological inhibitors such as amiloride, 9-phenanthrol and SKF96365, and in siRNA transfected rat PCA. The relationship between TRP channels and ENaC was also evaluated.

## Methods

This investigation was conducted in compliance with the *Guide for the Care and Use of Laboratory Animals* published by US National Institutes of Health (NIH publication No. 85–23, revised 1996). The experimental protocols used in this study were approved by the Ethics Committee and the Institutional Animal Care and Use Committee of Yonsei University, College of Medicine.

### Tissue preparation

Seventy adult male Sprague-Dawley rats were used in this study. Rats were anesthetized with sodium pentobarbital (50 mg/kg, i.p.). The depth of anesthesia was evaluated by pinching the animal's paw with forceps. Rat brains were isolated and placed in chilled normal Krebs-Henseleit (KH) solution composed of (in mmol/L): NaCl, 119; CaCl_2_, 2.5; NaHCO_3_, 25; MgSO_4_, 1.2; KH_2_PO_4_, 1.2; KCl, 4.6; and glucose, 11.1. PCAs were dissected from the brain, cleaned of connective tissue, and segmented into about 3–4 mm lengths. To eliminate the potential influence of endothelial factors on pressure-induced myogenic tone, an air bolus was passed through the lumen to disrupt the endothelium. PCA segments lacking a vasodilatory response to acetylcholine (1 µmol/L), but exhibiting efficient constriction to serotonin (10 µmol/L), were included in the studies.

### Measurement of myogenic tone using an arteriograph system

Rat PCA segments (100–250 µm inner diameter) were cannulated and mounted in a pressure myograph (Living Systems Instrumentation, Burlington, VT, USA) as previously described [19]. Briefly, the de-endothelialized cerebral arterial segments were stretched longitudinally to approximate *in situ* length and maintained at an intraluminal pressure of 40 mmHg for a 40 min equilibration period. After the equilibration period, the pressure was increased in a stepwise manner from 20 to 120 mmHg in 20-mmHg increments, and each pressure was maintained for 10 min to allow the vessel diameter to stabilize before measurements.

### Small interfering RNA transfection of cerebral arteries

Rat PCA was transfected with small interfering RNA (siRNA) as previously described [19]. Briefly, the segments were separated into four groups for transfection with non-targeting (NT) siRNA, βENaC siRNA, TRPM4 siRNA,or TRPC6 siRNA (Bioneer Inc., Daejeon, Korea). A final amount of 10 µg siRNA for βENaC, 20 µg (first pair 10 µg + second pair 10 µg) for TRPM4, or 20 µg (first pair 10 µg + second pair 10 µg) for TRPC6 was transfected with Lipofectamine™ 2000 (Invitrogen, Carlsbad, CA, USA) according to the manufacturer's instructions. siRNA duplexes were designed with RNAi designer software (Ambion, Austin, TX, USA) using accession number NM012648 for *β*ENaC, NM001136229 for TRPM4, and NM053559 for TRPC6. The NT siRNA was purchased from Bioneer Inc. The sequences of *β*EnaC siRNA duplexes were: forward, 5′-GGAGCUGCUAGUGUGGUACdTdT-3′; reverse: 5′-GUACCACACUAGCAGCUCCdTdT-3′. The first pair of sequences of TRPM4 siRNA were: forward, 5′-AGAGAGGAUCAUGACCCGAA-3′; reverse: 5′-UUCGGGUCAUGAUCCUCUCU-3′. The second pair of sequences of TRPM4 siRNA were: forward, 5′-CCUGGGUAACGUGGUCAGUUA-3′; reverse, 5′-UAACUGACCACGUUACCCAGG-3′. The first pair of sequences of TRPC6 siRNA were: forward, 5′-GAACGGCCUCAUGAUUAUUdTdT-3′; reverse, 5′-AAUAAUCAUGAGGCCGUUCdTdT-3′. The second pair of sequences of TRPP6 siRNA were: forward, 5′-GUGUACAGAAUGCAGCCAGdTdT-3′; reverse: 5′-CUGGCUGCAUUCUGUACACdTdT-3′. The sequences of the NT siRNA duplexes were: forward, 5′-CCUACGCCACCAAUUUCGUdTdT-3′; reverse, 5′-ACGAAAUUGGUGGCGUAGGdTdT-3′. Knockdown efficiency was assessed by western blotting.

### Immunofluorescence of γENaC, TRPM4, and TRPC6 proteins in PCAs

The expression and localization of γENaC, TRPM4, and TRPC6 proteins in isolated rat PCAs were measured using immunostaining as previously described [25]. The PCAs were probed with γENaC antibody (1∶200 dilution; Millipore, Temecula, CA, USA), TRPC6 antibody (1∶200 dilution; Abcam, Cambridge, UK), or TRPM4 antibody (1∶200 dilution; Abcam). DAPI was used for nuclear staining (Vector Laboratories, Burlingame, CA, USA).

### Western blotting

The expression of βENaC, TRPC6, and TRPM4 proteins in rat PCA was measured by western blot as previously described [26]. Membranes were probed with βENaC antibody (1∶200 dilution; Millipore), TRPM4 antibody (1∶200 dilution; Abcam), TRPC6 antibody (1∶200 dilution; Abcam), or actin antibody (1∶1000 dilution; Abcam) as the loading control. Bands were detected and quantified using Fuji Photo Film Image with the Fuji film image gauge program (version 2.54; Fuji Photo Film Co., Tokyo, Japan).

### Immunoprecipitation

Tissue homogenates were incubated with γENaC antibody overnight at 4°C. After incubation with protein G agarose beads for 2 h, precipitates were washed with lysis buffer and then resuspended in SDS-PAGE sample buffer. Samples were denatured at 100°C for 5 min prior to separation by SDS-PAGE.

### Drugs

The following drugs were used: amiloride (Tocris Bioscience, Ellisville, MO, USA); SKF96365 hydrochloride (Tocris Bioscience); 9-phenanthrol (Sigma-Aldrich, St Louis, MO, USA); and nifedipine (Sigma-Aldrich). The general laboratory reagents used were analytical grade or better.

### Statistics

All values given in the text are expressed as means ± SEM and were analyzed by two-way ANOVA, followed by Student–Newman–Keuls posthoc test. Differences were considered significant at the *P*<0.05 level.

## Results

### Localization of ENaC, TRPC6, and TRPM4 in rat PCA

**Figure 1:**
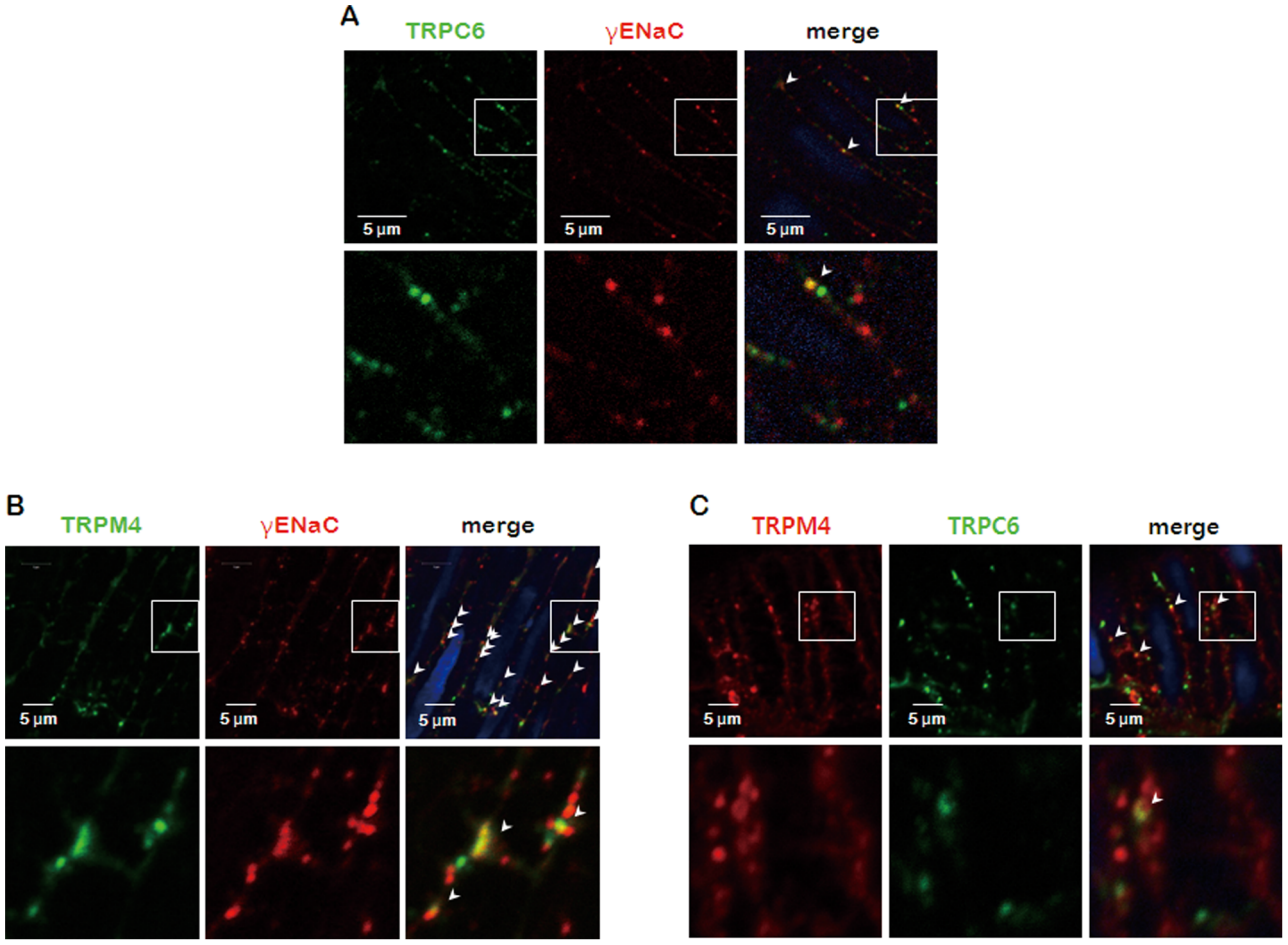
Localization of ENaC, TRPM4, and TRPC6 in rat PCA. A–C: Immunofluorescence of TRPC6 and γENaC (A), TRPM4 and γENaC (B), and TRPM4 and TRPC6 (C). The arrow and yellow coloration denotes co-localization of channels in merged images. Blue colors indicate the nucleus.

ENaC, TRPC6, and TRPM4 were expressed in rat PCA and typical immunofluorescent staining in PCA segments showed that ENaC, TRPC6, and TRPM4 were localized near the cell surface (Fig. 1A, B, & C).

### Effects of amiloride, SKF96365, and 9-phenanthrol on myogenic tone of PCA

**Figure 2:**
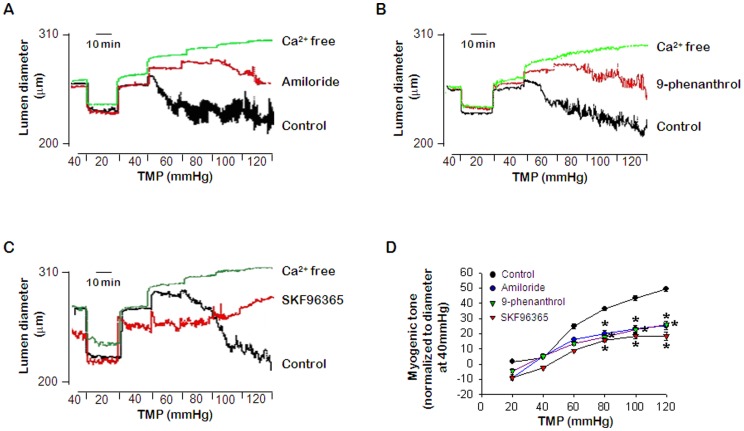
Effects of ENaC, TRPM4, and TRPC6 inhibition on the pressure-induced myogenic response. A–C. Representative recording showing the effect of 1 µmol/L amiloride (A), 5 µmol/L 9-phenanthrol (B), and 1 µmol/L SKF96365 (C), respectively. D. Mean data for the effect of amiloride, 9-phenanthrol, and SKF96365. Data are expressed as means ± SEM (*n* = 6) and are normalized to myogenic tone at a diameter of 40 mmHg. *p<0.05 for control vs. treatment with amiloride, 9-phenanthrol or SKF96365.

To determine whether ENaC, TRPC6, and TRPM4 proteins are required for the myogenic response, we evaluated myogenic tone in isolated PCA in the presence and absence of inhibitors. Figure 2A, B, and C indicate the typical traces showing effects of ENaC, TRPM4, and TRPC6 blockade on PCA, respectively. When intraluminal pressure was increased in a stepwise manner from 20 to 120 mmHg, the PCA contracted in response to an increase in intraluminal pressure, whereas, in Ca^2+^-free KH bath solution, vessels dilated passively in response to increases in intraluminal pressure. As shown in Figure 2, the myogenic tone in response to an increase in intraluminal pressure was inhibited by treatment with ENaC inhibitor amiloride (1 µM, Fig 2A) TRPM4 inhibitor 9-phenanthrol (5 µM, Fig 2B) and TRPC6 inhibitor SKF96365 (1 µM, Fig 2C). The myogenic tone was significantly inhibited by treatment of the inhibitors (Fig 2D) while resting diameter at 40 mmHg was not changed by the inhibitors (Fig. 2B, control, 257.5±26.16; amiloride, 224.22±17.55; 9-phenanthrol, 257.4±21.37; SKF96365, 317.00±25.51, respectively).

### Interactions among ENaC, TRPM4 and TRPC6: Effects of co-treatment with amiloride, SKF96365, and 9-phenanthrol on myogenic tone in PCA

To verify the interaction among ENaC, TRPC6, and TRPM4, we first performed immunofluorescence assay. To determine whether ENaC and TRPM4 are expressed in the same location, isolated vessel segments were co-stained with rabbit anti-γENaC and goat anti-TRPM4 antibodies. As shown in Figure 1B, γENaC and TRPM4 expression appeared to be clustered in similar locations within a smooth muscle cell in segmented PCAs. TRPC6 was located near the cell surface but was not co-localized with ENaC or TRPM4 (Fig. 1A & C). Similar data were obtained using immunoprecipitation and western blot assays. TRPM4 was immunoprecipitated from PCA homogenates by anti-ENaC antibody, but TRPC6 was not immunoprecipitated (data not shown).

**Figure 3:**
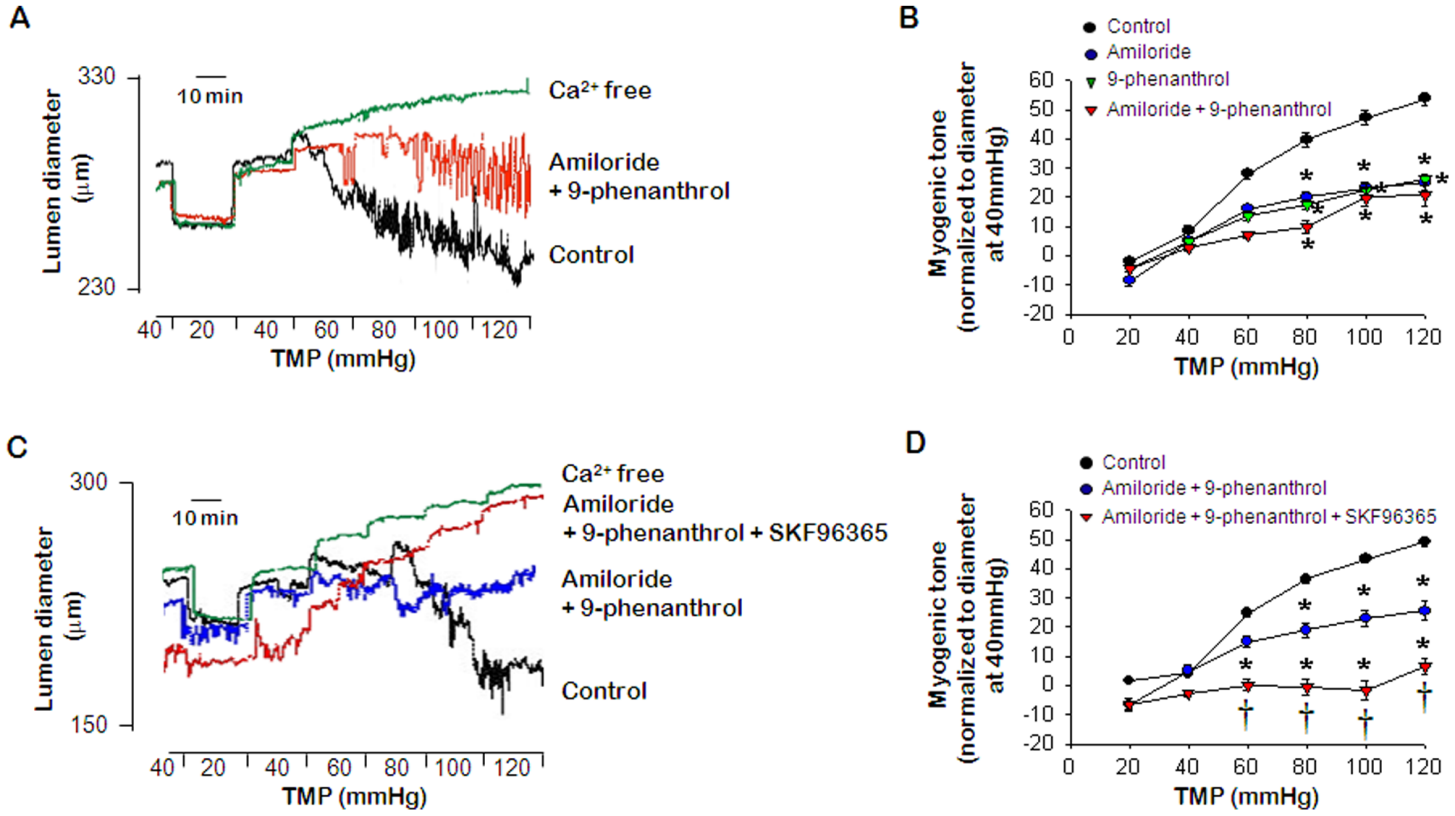
Effects of co-treatment with amiloride, SKF96365, and 9-phenanthrol on myogenic response. A. Representative recording showing the effect of co-treatment with 1 µmol/L amiloride and 5 µmol/L 9-phenanthrol. B. Mean data for the effects of amiloride, 9-phenanthrol, or both inhibitors. Data are shown as means ± SEM (*n* = 6) *p<0.05 for control vs. treatment with amiloride or 9-phenanthrol or co-treatment with amiloride and 9-phenanthrol. C. Representative recording showing the effect of SKF96365 on the myogenic response in PCA pretreated with amiloride and 9-phenanthrol. D. Mean data for the effect of SKF96365 on pressure-induced myogenic tone in PCA pretreated with amiloride and 9-phenanthrol. Data are shown as means ± SEM (*n* = 6). *p<0.05 for control vs. amiloride and 9-phenanthrol or amiloride, 9-phenanthrol and SKF96365. † *p*<0.05 for amiloride and 9-phenanthrol vs. amiloride, 9-phenanthrol and SKF96365.

The interaction between ENaC and TRPM4 suggested that ENaC and TRPM4 may have a second role in the regulation of the myogenic response. To test the biological significance of this interaction, we tested the effects of amiloride and 9-phenanthol on pressure-induced myogenic regulation. Inhibitors of ENaC and TRPM4 attenuated the pressure-induced myogenic response (Fig. 3A), but co-treatment with amiloride and 9-phenanthrol did not result in an additional effect on the inhibition of myogenic tone compared to single treatment with amiloride or 9-phenanthrol (Fig. 3B). These results suggested cross-talk between the function of ENaC and TRPM4. Co-treatment with all inhibitors fully inhibited the myogenic tone (Fig. 3C & D). As shown in Figure 3B, co-treatment with amiloride and 9-phenanthrol did not have additional effects on the myogenic tone compared to individual treatment with each inhibitor. On the other hand, the pressure-induced myogenic response was entirely inhibited by SKF96365 treatment in the PCAs co-treated with amiloride and 9-phenanthrol (Fig 3 C & D).

### Effects of ENaC, TRPC6, and TRPM4 siRNA on the protein expression and myogenic constriction in rat PCA

**Figure 4:**
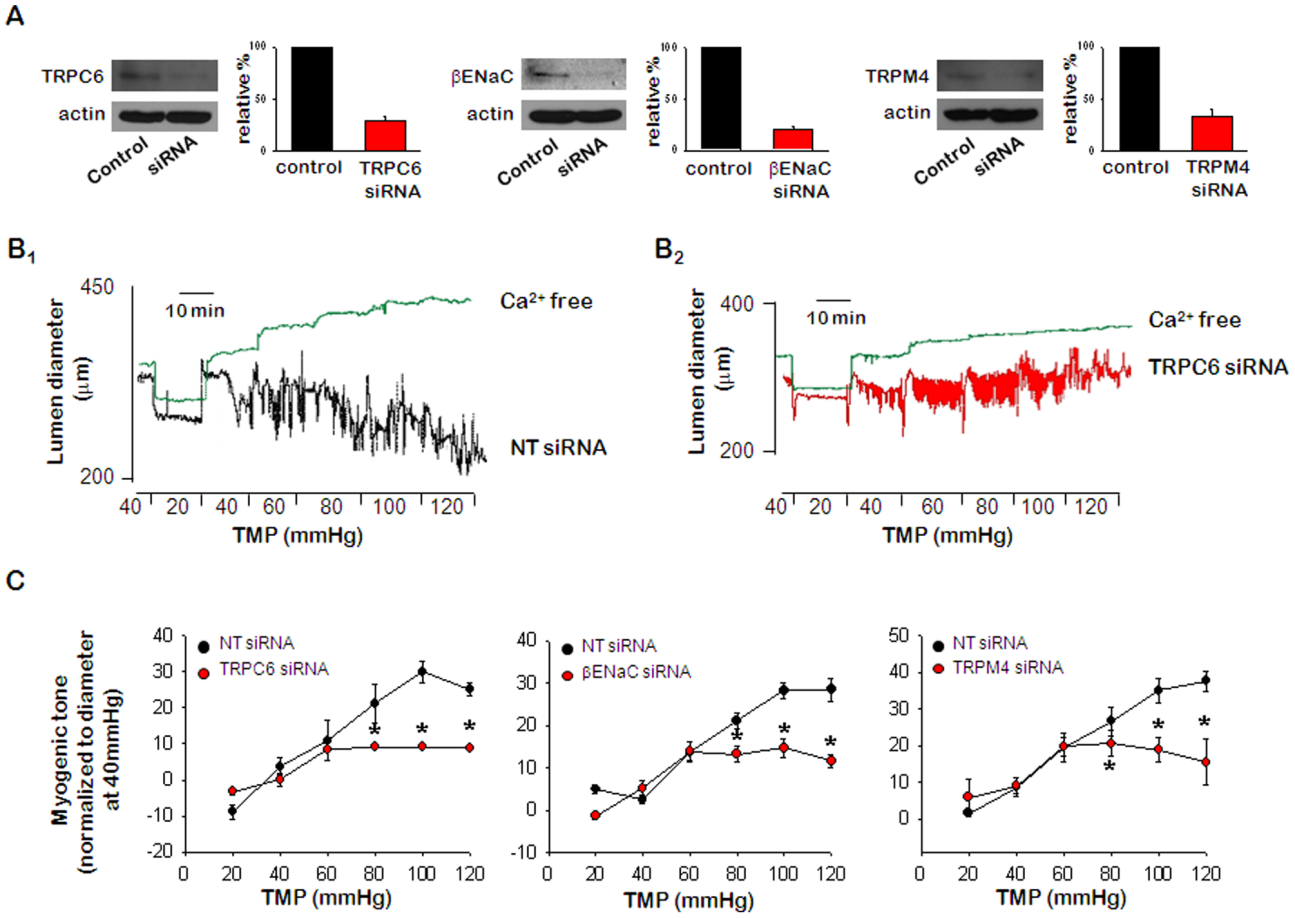
Effects of ENaC, TRPC6, and TRPM4 siRNA on the protein expression and myogenic response. A. Western blots showing the effect of TRPC6, βENaC, or TRPM4 siRNA on the expression of TRPC, βENaC, or TRPM4. Actin was used as a loading control. B. Representative recording of myogenic response in PCAs transfected with NT siRNA (B_1_) or TRPC6 siRNA (B_2_). C. Mean data for the effect of TRPC6-, βENaC-, and TRPM4-siRNA on pressure-induced myogenic tone. Data are shown as means ± SEM (*n* = 6). *p<0.05 for control (NT siRNA) vs. transfected with siRNA. TMP: transmural pressure.

The presence of ENaC, TRPC6, and TRPM4 proteins in PCA was confirmed by western blot analysis (Fig. 4A) and confocal microscopy (Fig. 1A, B, C). To obtain direct evidence for the role of ENaC, TRPC6, and TRPM4 in the development of myogenic tone, we transfected rat PCA with siRNA, a known post-transcriptional silencer. Transfection of ENaC siRNA reduced endogenous ENaC expression up to 80% when compared to non-targeting (NT) siRNA controls. In addition, TRPC6 and TRPM4 siRNA reduced endogenous expression of the respective proteins by up to 70% (Fig. 4A). We also investigated the effects of siRNA of ENaC, TRPC6, and TRPM4 on the pressure-induced myogenic response in rat PCA. As shown in Figure 4B and C, treatment with NT siRNA did not affect arterial contractility, but intraluminal pressure-induced vasoconstriction was inhibited in the PCAs transfected with targeting siRNAs (Fig. 4B_2_ & C). In this study, neither NT siRNA nor targeting siRNAs affected K^+^-induced constrictions (data not shown). As a result of these findings, we concluded that ENaC, TRPC6, and TRPM4 were all required for the myogenic response induced by an increase of intraluminal pressure.

### Interactions among ENaC, TRPM4 and TRPC: Effects of amiloride, SKF96365, and 9-phenanthrol on myogenic tone in ENaC, TRPC6, and TRPM4 siRNA transfected PCA

**Figure 5:**
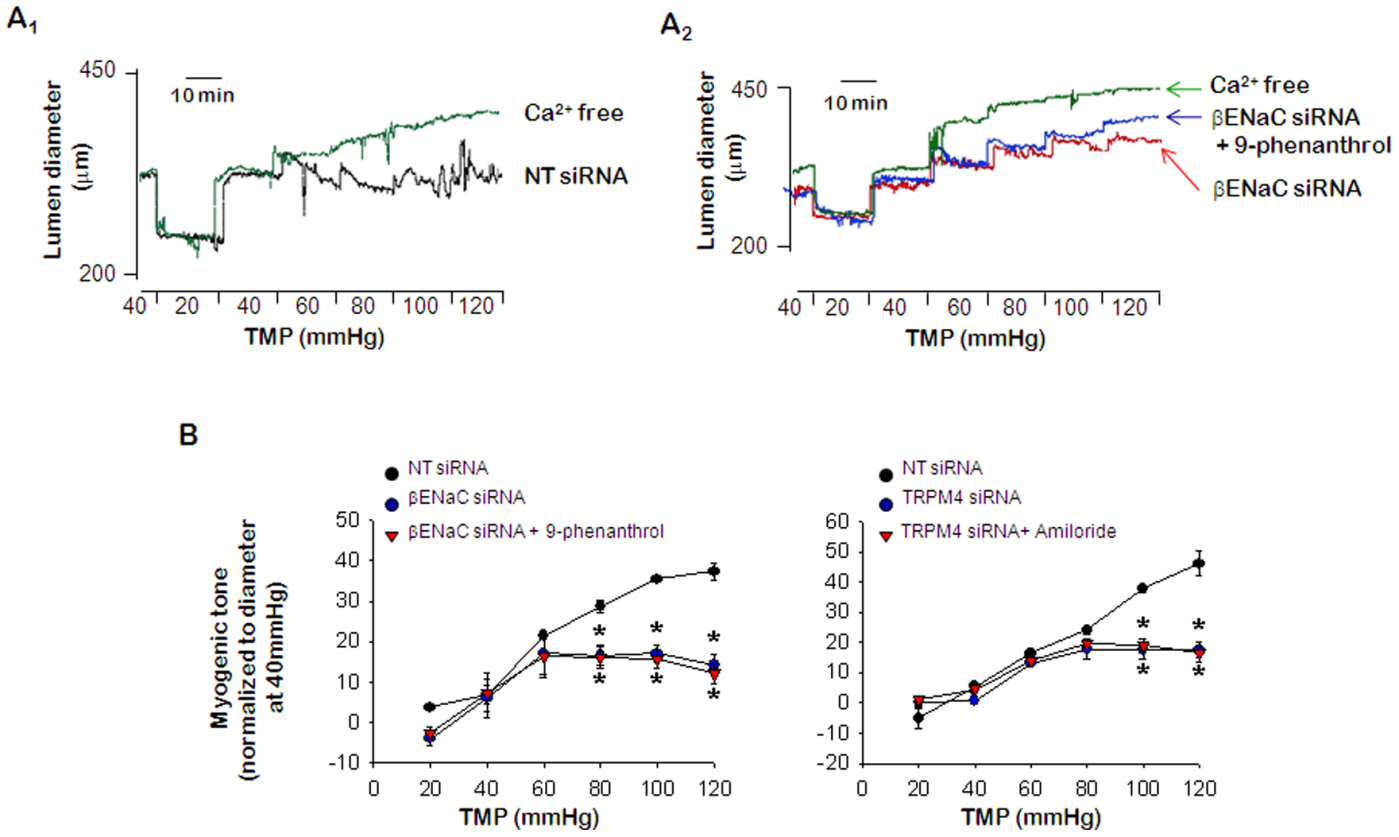
Effects of amiloride, and 9-phenanthrol on myogenic tone in ENaC, and TRPM4 siRNA transfected PCA. A_1_. Representative recording of myogenic response in PCAs transfected with NT siRNA. A_2_. Representative recording of the effect of 9-phenanthrol on myogenic tone in PCA transfected with βENaC-siRNA. B. Mean data for the effect of 9-phenanthrol or amiloride on the pressure-induced myogenic response in PCA transfected with βENaC or TRPM4 siRNA, respectively. Data are shown as means ± SEM (*n* = 6). *p<0.05 for control (NT siRNA) vs. transfected with siRNA, or control vs. transfected with siRNA and amiloride or 9-phenanthrol.

**Figure 6:**
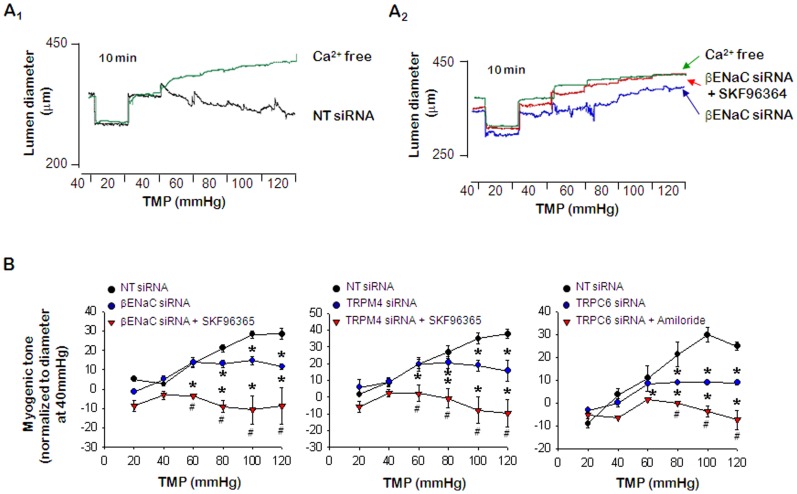
Effects of amiloride, and SKF96365 on myogenic tone in ENaC, TRPC6, and TRPM4 siRNA transfected PCA. A_1_. Representative recording of the myogenic response in PCA transfected with NT siRNA. A_2_. Representative recording of the effect of SKF96365 on myogenic tone in PCA transfected with βENaC siRNA. B. Summary of the effects of SKF96365 and amiloride on pressure-induced myogenic tone in PCA transfected with ENaC, TRPM4, or TRPC6 siRNA. Data are shown as means ± SEM (*n* = 6). *p<0.05 for control (NT siRNA) vs. transfected with siRNA or transfected with siRNA and amiloride or SKF96365. #p<0.05 for transfected with siRNA vs. transfected with siRNA and amiloride or SKF96365.

To verify the interaction among ENaC, TRPC6, and TRPM4, we examined the effects of inhibitors on myogenic responses in siRNA transfected PCA. In the inhibitor experiments, we detected co-treatment with amiloride and 9-phenanthrol did not have additional effects on the myogenic tone compared to individual treatment with each inhibitor (Fig 3B). Furthermore, we did not observe additional effects in βENaC siRNA transfected PCA treated with 9-phenanthrol (Fig. 5A_2_ & B) and in TRPM4 siRNA transfected PCA treated with amiloride (Fig. 5B). On the other hand, the pressure-induced myogenic response was entirely inhibited by SKF96365 treatment in the PCAs co-treated with amiloride and 9-phenanthrol (Fig. 3D), and transfected with βENaC or TRPM4 siRNA (Fig. 6A_2_ & 6B). Conversely, amiloride treatment in PCAs transfected with TRPC6 siRNA (Fig. 6B) also entirely inhibited the pressure-induced myogenic response. These findings suggested that TRPC6 did not interact with ENaC or TRPM4 in rat PCA.

## Discussion

The myogenic response is characterized by a decrease or increase of the vessel diameter in response to changes in transmural pressure, and plays an important role in the maintenance of ambient vascular tone and the autoregulation of blood flow of the resistance vasculature. Thus it prevents transmission of high systemic pressure to the fragile microvasculature [27]. Furthermore, the myogenic response has been considered as an important factor especially in cerebral circulation because cerebral arteries are not particularly responded to the sympathetic nerves surrounding them [28].

It has been also reported that impaired cerebral arterial function may be responsible for the development of cerebral injury and edema that can progress to eclampsia, and the myogenic response is an important auto-regulatory mechanism to control cerebral arterial function [29], [30]. Previous studies in nematodes, flies, and mammalian hair cells have led to the development of mechanosensory models that provide insight into how VSMCs sense and transduce mechanical signals [31]–[34]. Although the signal transduction pathways underlying VSMC contraction have been investigated, how the increase in pressure triggers vasoconstriction has remained unclear. Numerous structural elements have been proposed as participants in the transduction of stretch into cellular signaling events in VSMCs, including: 1) membrane-bound enzyme and second messenger systems, 2) activation of ion transporters and exchangers, and 3) mechanosensitive ion channels [2], [27]. Recently, there have been several reports about the requirement of ENaC, TRPC6, and TRPM4 proteins for myogenic constriction [13], [14], [16], [17], [35], [36]. These reports have demonstrated the expression of ENaC proteins and the effect of blocking ENaC protein with amiloride on myogenic constriction [14], [17]. Downregulation of TRPC6 or TRPM4 expression using antisense oligodeoxynucleotides has been shown to abolish pressure-induced depolarization and constriction in isolated cerebral arteries [13], [16], [35]. Previous studies from our laboratory also demonstrated that inhibition of ENaC abolishes pressure-induced vasoconstriction in PCAs [19]. In this study, we demonstrated for the first time that TRPM4 and TRPC6 as well as ENaC appeared to be necessary for myogenic constriction in rat PCAs. We also found that ENaC is functionally linked to TRPM4, but not to TRPC6. We demonstrated that ENaC, TRPM4, and TRPC6 proteins were expressed in rat PCA using immunostaining and western blot analysis. Our study revealed that ENaC, TRPM4, and TRPC6 proteins were expressed and localized near the membrane of vascular smooth muscle cell (Fig. 1A–C & Fig. 4A). These results are consistent with previous research [13], [16], [35]. The localization pattern is important since the cell membrane is the site where mechanosensors could be located.

To determine the role of ENaC, TRPM4, and TRPC6 in the myogenic response, we evaluated the effects of blocking these channels on the pressure-induced myogenic response in rat PCA. Two different approaches, inhibitors and siRNA transfection, were used to investigate whether these channels were required for myogenic constriction. Similar to the results from previous reports [14], [37], we found that myogenic constriction was inhibited by amiloride, the ENaC inhibitor (Fig. 2). Pressure-induced vascular tone was also inhibited by treatment with TRPM4 inhibitor 9-phenanthrol [38], [39], or canonical TRPCs inhibitor SKF96365 [40], [41]. The high K^+^-induced vasoconstriction was not affected by the inhibitors (data not shown), which means vascular reactivity was not affected by the inhibitors. The concentrations of inhibitors applied in our study are half-maximal effective concentrations (EC_50_). And these doses are also chosen by several researchers in the previous studies [42]–[44]. Although the inhibitors of these channels attenuated the pressure-induced myogenic response, the results could not directly prove that ENaC, TRPC6, and TRPM4 proteins function as mechanosensors. In contrast, knock-down strategies provide strong evidence for the important functional role of channel subunits in vascular mechanotransduction. Thus, to strengthen our data, we used a siRNA approach to reduce the expression of these channels in cerebral arteries. We showed that βENaC, TRPM4, and TRPC6 siRNA were effective to reduce protein levels by 70–80% after 3 days of transfection (Fig. 4A). siRNA transfection did not affect the high K^+^-induced constriction (data not shown), but vasoconstriction induced by elevating intraluminal pressure was inhibited almost 50% in arteries transfected with siRNAs compared to NT-siRNA-transfected arteries (Fig. 4C). Our results demonstrated that ENaC, TRPM4, and TRPC6 proteins play important roles as mechanotransducers in the myogenic response. Furthermore, we found that myogenic tone was decreased by ∼50% in the presence of amiloride, 9-phenanthrol and SKF96365 (Fig. 2B).

There has been no evidence that ENaC and TRP channels are directly or indirectly linked. We therefore performed immunoprecipitation and western blot assays to demonstrate binding of TRP channels to γENaC. We found that TRPM4, but not TRPC6, coimmunoprecipitated with γENaC from PCA homogenates (data not shown). Moreover, TRPM4 and γENaC co-localized in the apical membrane of arterial smooth muscle cells as visualized by laser-scanning confocal microscopy (Fig. 1B). TRPC6 was also expressed at the smooth muscle cell membrane, but was not co-localized with γENaC or TRPM4 (Fig. 1A & 1C). We also found that actin interacted with γENaC, as previously reported [41]. We speculated that ENaC interacted with TRPM4 and actin, which is part of a large, multicomponent protein similar to the established model of the mechanosensors in VSMCs [27].

Pressure-induced myogenic tone was inhibited by treatment with amiloride or 9-phenanthrol, but co-treatment with the inhibitors did not have additional effects on inhibition of the myogenic response compared to single inhibitor treatment (Fig. 3A & 3B). Furthermore, we found that treatment with 9-phenanthrol or amiloride had similar effects to siRNA transfection of βENaC or TRPM4 on the myogenic response in PCA (Fig. 5B). These results suggested that ENaC could be functionally linked with TRPM4. In addition, these data were in agreement with immunoprecipitation and confocal microscopy findings.

Our findings, for the first time, suggested a relationship between ENaC and TRP channels in intact resistance arteries. We demonstrated that ENaC, TRPM4, and TRPC6 were required for the development of myogenic response induced by an increase in intravascular pressure. Co-treatment with amiloride, 9-phenanthrol and SKF96365 fully inhibited the pressure-induced myogenic tone (Fig. 3C & 3D), and SKF96365 had an additional inhibitory effect on the myogenic response in βENaC or TRPM4 siRNA transfected PCA (Fig. 6B). Moreover, the myogenic response was fully suppressed by amiloride treatment of TRPC6-siRNA-transfected PCA (Fig. 6B). These results consistently indicated that TRPC6 did not interact with ENaC or TRPM4 in rat PCA.

**Figure 7:**
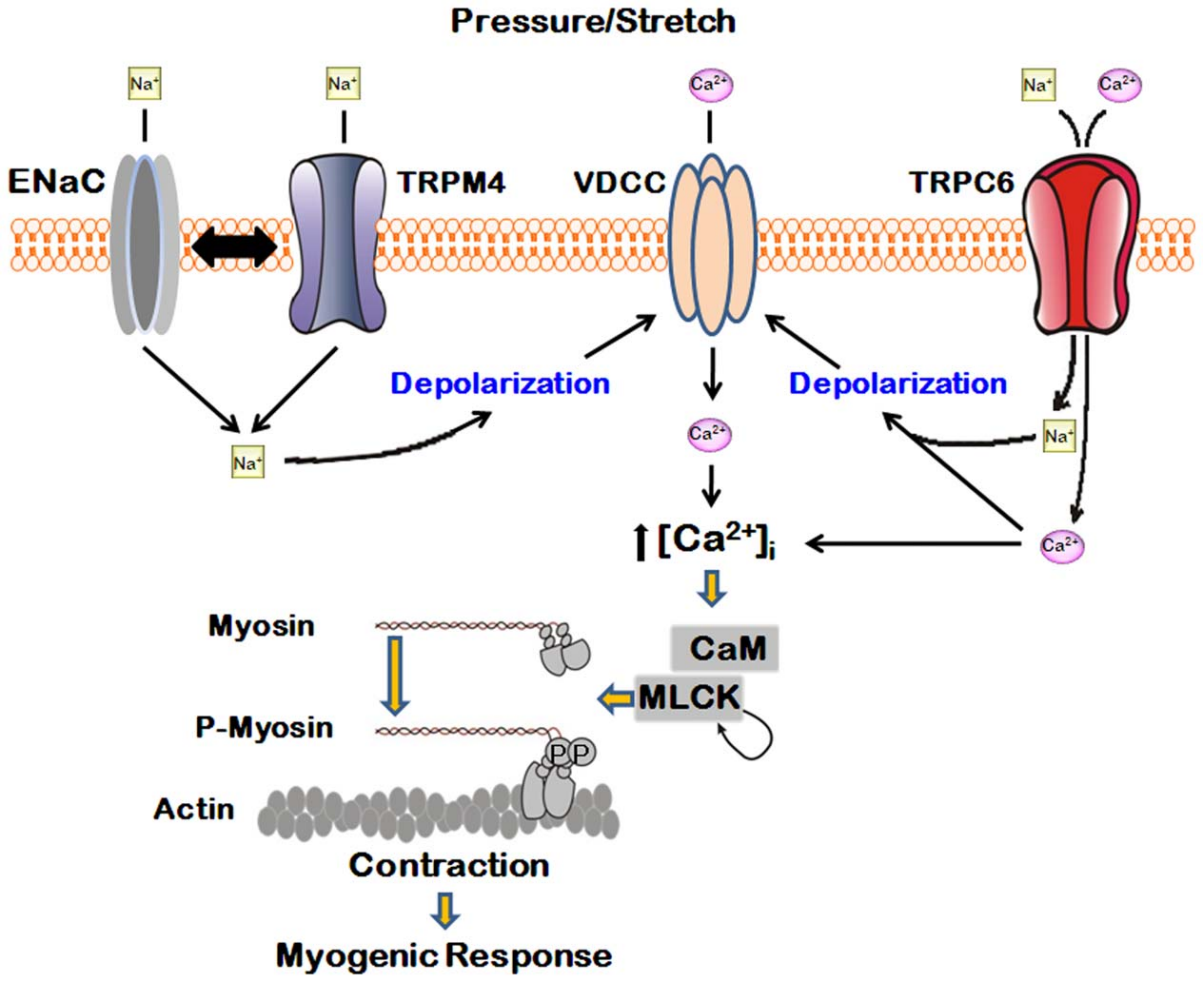
Summary for the role of mechanosensitive ion channels in myogenic tone development. Pressure or stretch activates ENaC, TRPM4, and TRPC6. Activation of these channels leads to induce depolarization and subsequent activation of voltage dependent Ca^2+^ channel (VDCC). As a result, intracellular Ca^2+^ level is elevated and myogenic response is induced.

In summary, pressure or stretch induces activation of mechanosensitive ion channels and then membrane depolarization and subsequent Ca^2+^ influx via voltage dependent Ca^2+^ channels (VDCC) largely mediates myogenic contraction (Fig. 7). Furthermore, our findings point to roles of ENaC, TRPM4, and TRPC6 as sensors of mechanotransduction during the myogenic response induced by an increase in intraluminal pressure. We postulate that ENaC is functionally linked to TRPM4.
